# Unsafe Injection Practices by Medical Practitioners in South Asia Associated with Hepatitis and HIV Outbreaks

**DOI:** 10.29245/2689-9981/2018/2.1113

**Published:** 2018-08-01

**Authors:** Arshad Altaf

**Affiliations:** World Health Organization, Western Pacific Region, Manila, Philippines

Injection is one of the most common medical procedures in the world. Most medical injections are provided for curative reasons whereas some of the other reasons include vaccination injections, family planning and diabetic injections^1^. A safe injection does not harm the recipient, does not expose the provider to risk and does not result in waste that is dangerous for others^2^. Observing infection prevention and control procedures and use of a single injection equipment markedly reduces the risk of transmission of infection. Unsafe injection practices include an injection given with a syringe used on more than one patient, injections prepared in unclean area compromising infection prevention and control practices and injections given unnecessarily in conditions that can be treated with oral medicines for example common flue or minor aches and pains. Unsafe injections lead to transmission of blood borne infections including hepatitis B virus (HBV), hepatitis C virus (HCV) and human immuno deficiency virus (HIV)^3^. A mathematical modelling study estimated that unsafe injections in health care settings transmit 1.67 million HBV infections, 315,210 HCV infections and 33,877 HIV infections^4^. In most medical situations an injection is provided by a front-line health care worker who could be a nurse or another trained person like a para medic (often called a dispenser or a compounder in South Asia) or a nurse assistant while in some situations a doctor also provides the injection. While unsafe injection practices rarely occur in high income countries, but they are often evident in low income country health settings where the administration of injection takes place in a variety of settings and involve a range of providers which could be trained or untrained, traditional healers and sometime relatives and neighbors providing an injection as a favor or expression of care^5^. A review on unsafe injections from South Asia pointed out that 5% to 50% of injections are provided with reused syringes, thus creating potential to transmission of blood-borne pathogens. Qualified and unqualified practitioners, especially in the private sector, are the major drivers behind injection use, but patients also prefer injections, especially among the rural, poor or uneducated in certain countries^6^.

In February 2018 in South Asia, two HIV outbreaks have been linked with unsafe injection practices and breaks in infection control practices. In Unnao, Uttar Pradesh, India, 21 people were infected with HIV after an unqualified practitioner used a syringe more than once on multiple patients to administer injections. The issue came into authorities’ attention when they started observing a high number of HIV cases from a particular area. Relevant health authorities screened 566 people for HIV out of which 21 were confirmed HIV positive^7^. The transmission was traced to an unqualified practitioner. The HIV positive patients were referred to nearby antiretroviral therapy center. The police reportedly arrested the practitioner. He was found to be providing door to door cheap medical service to the villagers^8^. The same month, 22 people in Sargodha district of Punjab, Pakistan were confirmed HIV positive in Kot Imrana town. The health department had screened 80 persons and 22 were tested positive for HIV^9^. In this situation also, unsafe injections were found to be the most probable cause. It is important to mention that these were initial reports and suffer from multiple epidemiological weaknesses while making a firm association between infection and disease transmission. While a formal outbreak investigation usually ensues and is published some years later, but it is prudent that these issues are highlighted.

Transmission of HBV, HCV and HIV due to poor infection control and unsafe injection practices have previously been reported from South Asia region. In 1997 following an unusually high number of hepatitis C cases in Hafizabad, Pakistan a matched case control study found that compared to non-infected controls, HCV cases were significantly more likely to have received 5 or more injections in the previous 10 years (odds ratio, 5.4; 95% confidence interval, 1.2-28.0). Only 5 HCV cases had received blood transfusions, and none reported intravenous drug use^10^. A case control study in 2003 enrolled acute cases of HBV and controls from four hospitals and assessed exposure to risk factors for HBV infection. Cases in this study were more likely to have received one or more than one injection during the time window considered for exposure. The population attributable risk for therapeutic injections was 53%^11^. Another cross-sectional study in 2010 identified risk factors for hepatitis C in a peri urban community in Karachi, Pakistan where HCV was found to be as high as 23.8% among 1997 study participants. Among women, unsafe health care practices, while among men extramarital sex, shaving from a barber and hospitalizations were associated with HCV infection^12^. In 2008, in Gujrat, Pakistan an HIV outbreak was investigated in which 53 HIV infected persons were investigated by the Pakistan Field Epidemiology Training and Laboratory Program (FELTP). Out of 47 alive cases at the time of investigation there were 27 females and six children under 10 years or less. The investigation identified exposure to unsafe injections, dental procedures and barber shop visits as the key risk factors^13^.

An injection practice assessment study from southern part of India in 2003 concluded that there were deficiencies in practice such as an excessive, unwarranted usage of injections, a sizeable prevalence of unsafe injection practices, the short supply of injection equipment leading to a high incidence of needlestick injuries, a low proportion of hepatitis B virus immunization among providers, and a lack of adequate sharps containers and disposal facilities^14^. In 2009 an outbreak of hepatitis B was reported from Gujarat, India in which 664 (77.57%) out of 856 hospitalized cases belonged from one district. Epidemiological and laboratory investigation followed, and government authorities confirmed that unsafe injections by private practitioners was the main reason of the outbreak^15^. In State of Punjab, India, the high prevalence of hepatitis C (5.2%) and associated risk factors were identified as unsafe medical practices (including unsafe injections and dental procedures) and intravenous drug use^16^. Qualitative data from the national assessment in India suggest that injections are prescribed by private practitioners for money, for quick relief, and to maintain credibility among patients^17^.

Like HIV, HBV and HCV are spread by sharing needles, syringes, and other injection equipment. Both viruses can also be transmitted sexually, but HBV is much more likely than HCV to be transmitted sexually. Sexual transmission of HCV is most likely to happen among gay and bisexual men who are living with HIV. Pregnant women can pass these infections to their infants^18^.

It is worth mentioning that increased access to highly effective direct-acting antivirals (DAAs) for the treatment of infection with HCV is revolutionizing the prospect of ending HCV epidemics. Globally, the number of people who initiated DAA-based treatment for HCV rose between 2015 and 2016, from approximately 1 million to 1.5 million. A small number of countries were responsible for the bulk of that increase. Egypt and Pakistan accounted for about half of all people who started DAA treatment in 2016^19^.

HIV attracts attention due to the nature of the illness. As a knee jerk reaction after an HIV outbreak a short awareness campaign is launched with the objective to educate the masses. The health advertisements highlight the dangers of HIV or hepatitis infections and educate about risk factors. But this is not sufficient.

Injection safety is a complex problem and requires a multi modal approach. The World Health Organization in 2015 launched injection safety guidelines (http://apps.who.int/iris/bitstream/10665/250144/1/9789241549820-eng.pdf) and one of the key recommendations was use of safety engineered syringes by all Member States by 2020. The other WHO recommendations include improving monitoring and regulation, training of health workers, educating the communities and appropriate sharp and health care waste management.

A review article on injection safety in 1999 had recommended that, “making a substantial reduction in the number of unnecessary injections may not be possible for many years and it may be more useful to aim to reduce unsafe administration. People may be more willing to take measures to ensure safety than to give up a potent symbol of optimal care”^20^. Approaches and interventions suitable to each country’s situation needs should be tested and implemented. However, adhering to good infection prevention and control practices, use of single use injection equipment, strengthening the health care delivery system in addition to other interventions such as safer sex practices, vaccination for hepatitis B and creating awareness of methods to reduce the risk of transmission among the masses are some of the approaches to prevention of HIV and hepatitis outbreaks.
